# Gravity and active acceleration limit the ability of killer flies (*Coenosia attenuata*) to steer towards prey when attacking from above

**DOI:** 10.1098/rsif.2021.0058

**Published:** 2021-05-26

**Authors:** S. Rossoni, S. T. Fabian, G. P. Sutton, P. T. Gonzalez-Bellido

**Affiliations:** ^1^ Department of Zoology, University of Cambridge, Cambridge CB2 3EJ, UK; ^2^ Department of Physiology, Development and Neuroscience, University of Cambridge, Cambridge CB2 3EL, UK; ^3^ School of Life Sciences, University of Lincoln, Lincoln LN6 7TS, UK; ^4^ Department of Ecology, Evolution and Behaviour, University of Minnesota, Saint Paul, MN 55108, USA

**Keywords:** diptera, drag, flight kinematics, proportional navigation, predation

## Abstract

Insects that predate aerially usually contrast prey against the sky and attack upwards. However, killer flies (*Coenosia attenuata*) can attack prey flying below them, performing what we term ‘aerial dives'. During these dives, killer flies accelerate up to 36 m s^−2^. Although the trajectories of the killer fly's dives appear highly variable, proportional navigation explains them, as long as the model has the lateral acceleration limit of a real killer fly. The trajectory's steepness is explained by the initial geometry of engagement; steep attacks result from the killer fly taking off when the target is approaching the predator. Under such circumstances, the killer fly dives almost vertically towards the target, and gravity significantly increases its acceleration. Although killer flies usually time their take-off to minimize flight duration, during aerial dives killer flies cannot reach the lateral accelerations necessary to match the increase in speed caused by gravity. Since a close miss still leads the predator closer to the target, and might even slow the prey down, there may not be a selective pressure for killer flies to account for gravity during aerial dives.

## 1. Introduction

Aerial predation is an intricate task, in which the predator's interaction with gravity changes according to the angle of its flight path. When flying upwards, predators need to overcome the force of gravity, but when chasing prey towards the ground, gravity will increase the total acceleration they achieve. Many birds of prey exploit gravity, using it to gain most of their velocity [1–5]. The opposite hunting strategy consists of perching close to the ground and looking upwards, which makes targets easily detectable against a clearer background, the sky. This strategy is common among predators with limited visual resolution such as insects, like many dragonflies and some robber flies [6,7].

When attacking aerial prey, dragonflies [6], hoverflies [8] and robber flies [7,9] fly towards the future location of their prey, i.e. they employ interception. To intercept their target, predatory dipteran flies are thought to use a strategy called proportional navigation (PN) [7]. Under PN, the predator monitors the change in the bearing to the prey and uses this information to correct its own heading. This law allows an animal to intercept a target without knowledge of the target's absolute distance or velocity. Another strategy is to head towards where the target is currently perceived, which guarantees a capture only if the chaser is faster than its prey. This strategy, known as pursuit, is employed by tiger beetles [10], house flies [11,12], long legged flies [13] and honeybees [14]. Of all the predatory insects studied thus far, none has been reported to attack downwards, if not for a small portion of the chase.

The fact that most predatory insects have not evolved to hunt downwards suggests that the challenges posed on their visual system are not counterbalanced by substantial advantages. The potential contribution of the force of gravity to their speed is likely heavily counteracted by air resistance, i.e. drag. Viscous drag scales with the projected body area, as inertia does with mass. Thus, the movement of a flying insect is influenced by both inertia and viscous drag, unlike the heavier vertebrates, whose movement is mainly dominated by inertia. Quantitatively, the ratio of inertial to viscous forces is represented by the Reynolds number. Flying insects operate at intermediate Reynolds numbers (1–10^3^), making them extremely useful investigative tools in biophysics [15,16].

As explained above, the large drag forces experienced by a downward-flying predatory insect likely reduce the relative contribution of gravity to its velocity. Meanwhile, the sensory complexity of detecting prey against the cluttered ground can be considerable. For these reasons, one may predict small predatory insects with low visual acuity to contrast prey against the sky and attack upwards. However, the killer fly *Coenosia attenuata*, a 4 mm long dipteran whose retina has a relatively poor spatial resolution of 2.2° [17], does not conform to such an expectation. Killer flies are fast and highly manoeuvrable dipteran predators, who hunt prey flying downwards as well as upwards [18]. In addition, in an enclosed arena, we observed killer flies positioning themselves on the arena ceiling and then attacking prey passing below them from such inverted position. Although not strictly typical of the killer fly's ecology in the wild, this behaviour provides a unique opportunity to investigate how aerial predators at intermediate Reynolds numbers are affected by gravity.

When hunting prey flying across from them or above them, the killer flies' trajectories are well explained by PN [7]. However, before the present paper, the killer fly's control system during downward attacks and whether killer flies account for gravity when generating vertical lift remained to be investigated. In this article, we compare attacks from the walls and floor of the behavioural arena to attacks from the arena ceiling, which we call dives. We analyse the dive kinematics and test the predictive power of PN and other steering models. After identifying a candidate navigational model, we test whether killer flies time their take-off to deploy an attack of minimal duration, or an attack that requires minimal power. Finally, we contextualize the killer fly's dive in the ecological relevance of it and its prey.

## 2. Material and methods

### 2.1. Animals

Female killer flies (*Coenosia attenuata*) [19] were taken from a laboratory colony, established from animals collected in Almeria (Spain). The colony was kept at 60–70% humidity and a 20–25°C temperature in a 12/12 hour light/dark cycle, and was fed live fruit flies (*Drosophila melanogaster*).

### 2.2. Animal preparation

Killer flies were individually placed in transparent vials with only wet filter paper provided for 2–3 days before testing, to ensure the animals were motivated to hunt [18]. After isolation, two flies were tested in the arena at a time, at room temperature of 20–22°C. The arena was a transparent 160 × 160 × 300 mm box made of 4 mm wide acrylic boards (Perspex^®^, Mitsubishi Chemical Lucite Group Ltd, Tokyo, Japan), placed with the long axis resting on a white table. The arena was illuminated with two artificial light sources (4 Long-Studio, VelvetLight, Barcelona, Spain) each producing an output of 14 000 lx at 1 m, comparable to peak light levels of a moderately cloudy day [20].

After placing the flies in the arena, a target was moved along the longitudinal dimension of the box, perpendicular to gravity, in either direction. The target was a black 2.1 mm bead, moved between 0.65 and 0.8 m s^−1^ [18] on a transparent 0.15 mm thick fluorocarbon fishing line (Vanish, Berkley Fishing, IA, USA). The line ran along pulleys fixed at the corners of a transparent acrylic support and was moved by a 23HS-108 MK.2 stepper motor controlled through computer software by an ST5-Q-NN DC motor controller (Applied Motion Products, Watsonville, CA, USA). The target always started moving outside the box. Flies were tested on repeated trials until they stopped responding to the target.

In order to measure the velocity of killer flies in free fall, female animals were taken from the colony and anaesthetized with carbon dioxide. While unresponsive, each fly was dropped from a height of 160 mm, the same height of the behavioural arena.

To extract morphological measurements, female killer flies were also sedated with carbon dioxide. While unresponsive, they were weighed (sensitivity: 0.1 mg), before having both their wings clipped and photographed in pairs for area measurements. Before clipping, a female killer fly was photographed, with its wings extended anteriorly and then posteriorly, to approximate the maximum stroke angle.

### 2.3. Photography, videography and data extraction

We used two time-synced SA2 Photron cameras (Photron Ltd, Tokyo, Japan) for filming. The recording frame rate used to digitize trajectories was 1000 frames/second, while the frame rate to extract wingbeat frequency was 2500 frames/second. The system was calibrated using an altered version [9] of the J.Y. Boguet's Laboratory's Matlab toolbox (Caltech, http://www.vision.caltech.edu/bouguetj/calib_doc/), running on Matlab R2014a (v. 8.3, MathWorks Inc., Natick, MA, USA). The movements of the killer flies and target were digitized offline using custom Matlab scripts for supervised automatic tracking [9], and smoothed through a fitting algorithm combining trajectory generation using linear fitting with jerk minimization [21], run on Matlab R2012a (v. 7.14).

Wingbeat frequency was calculated directly from the videos recorded at 2500 frames/second, as the reciprocal of the time taken between two consecutive wing supinations [22]. The photographs of clipped wings were analysed in ImageJ (v. 2.1.0/1.53c, National Institutes of Health, Bethesda, MD, USA). We fitted a polygonal shape to the wing in the pair with the sharpest edges, using the inbuilt tool, and then measured the wing area. We also used the inbuilt angle tool to measure the wing angle relative to the body when extended anteriorly and posteriorly (electronic supplementary material, figure S1). The angle difference approximated the maximal stroke amplitude.

### 2.4. Dynamics analysis

Kinematic measurements were performed from smoothed trajectories, analysed offline using Matlab R2018a (v. 9.4). First, we quantified the trajectory angle of the active hunts. To calculate this angle, we took the initial portion of the killer fly's trajectory, from take-off to the first point of minimal distance to the target's trajectory. The killer fly's trajectory was fitted with a line using the method of least squares. The trajectories were then projected on a plane orthogonal to the take-off surface and crossing the target trajectory. The trajectory angle was calculated as the angle between the fitted line and an axis orthogonal to the take-off surface. Therefore, more horizontal dives were assigned higher angles and more vertical dives were assigned lower angles.

For each recorded trajectory, we calculated the three-dimensional vectors of velocity and acceleration as the first and second derivative of object position. The acceleration of gravity (9.8 m s^−2^) was then subtracted from the vertical component of the total acceleration. The remaining three-dimensional acceleration was multiplied by the average fly mass, to obtain the flight force. Power was then calculated as the dot product of the flight force and the fly's velocity at each time point. For comparison with previous literature, we divided the power by the fly's flight muscle, to calculate the power density. To do this, we assumed 30% of the fly's mass to be devoted to flight muscle, as found in other *Schizophora* [23,24]. We also calculated the aerodynamic wing power densities (profile power, *P*_pro_, and induced power, *P*_ind_). To do this, we identified correlations in the literature [23] between aerodynamic power and flight force, normalized by body weight. We redigitized the data reported and fitted a linear regression for both profile power and induced power. We then used the killer flies' flight force magnitude, divided by the value of their body weight, to find *P*_pro_ and *P*_ind_ through the fitted regressions. The aerodynamic power, *P*_aero_, was then calculated as the sum of the two aerodynamic powers.

Also for comparison, we approximated the Reynolds number (*Re*) for diving killer flies' wings [25]:

2.1 $$Re=\;\frac{2S}{\upsilon }n\Phi ,$$

where *S* is the mean wing surface area, *υ* is the kinematic viscosity of air at 20°C (1.53 × 10^−5^ m^2^ s^−1^ [26]), *n* is the mean stroke frequency during dives and *Φ* is the maximum stroke amplitude.

### 2.5. Steering model selection

All flight simulations were run in Matlab R2018b (v. 9.1). The initial 5% of each hunt was discarded from simulations to account for irregularities of flight accelerations linked to the animals getting airborne. At the first time point of the simulations, the models were fed the target's position and the killer fly's position and velocity. Subsequently, simulations were only fed the target's relative position, used to calculate the steering, and the killer fly's relative linear acceleration, so that both killer fly and simulation had identical speed. In this study, we compared the steering dictated by pursuit to the steering of PN. We excluded models that require knowledge of absolute target size and speed (electronic supplementary material, table S1), because killer flies do not appear to have such knowledge [18].

A pure pursuit system minimizes the error (*δ*) between its heading and the virtual line connecting it to its target, called the line-of-sight (LOS). The controller thus induces a steering $\dot{\gamma }$ which corrects this error *δ* by an intrinsic constant *k*, according to formula:

2.2 $$\dot{\gamma }=k\cdot \delta .$$

After testing for a range of biologically plausible values (1–100 s^−1^; see electronic supplementary material, figure S2), the intrinsic constant *k* was fixed at 20 s^−1^ for comparative purposes, as used in previous work on the killer fly [7] and similar to the value measured in the lesser house fly [11].

PN uses the rotation of the LOS to the target to proportionally rotate the pursuer's heading [27]. The model takes the form:

2.3 $$\dot{\gamma }=N\cdot \dot{\lambda },$$

in which $\dot{\gamma }$ is the rotation of the pursuer heading, is the rotation of the LOS and *N* is a fixed gain, termed the navigation constant. After testing for a range of values (see results, §b), the navigation constant for simulations was fixed at *N* = 1.3, with a 20 ms lag between stimulus and response, matching the best fitting values from constant value sweeps from diving trials.

To test the performance of pursuit and PN in replicating the trajectories of hunts from the ceiling, we compared the axis of the heading rotation required by each steering control to the heading of real flies. In planar situations, these axes are either aligned or directly opposed. However, during 3D chases, the axes required of PN and pursuit do not necessarily align. Comparing the heading rotation of the models to real flies has the advantage of revealing inconsistencies even in the case of added modulators in the pursuit paradigm (equation (2.2)), such as the derivative or integral of the error *δ* proposed in other systems [11]. The steering controller which could best predict the real animals' flightpaths, PN, was then used for the remainder of the computational work.

To check for trajectory optimality, we used the range vector correlation [7,28]. We first calculated the vectorial difference between consecutive LOS and then extracted the angle between this difference vector and the corresponding LOS. The angle was then converted to a correlation value. During interception, collision is guaranteed for vector range correlations of −1, which indicate that LOS are parallel and getting shorter; however, values below 0 can still lead to interception.

Steering was limited to reflect hypothesized biological limits. The steering limitation was generated by fixing the maximal amount of lateral acceleration, i.e. the component of acceleration orthogonal to velocity, that the model could apply to the simulated fly. Lateral acceleration (*a*_lat_) was calculated in the manner below:

2.4 $$|a_{\mathrm{tang}}|=a\cdot \hat{v\;}$$

2.5 $$a_{\mathrm{tang}}=\;\hat{v\;}|a_{\mathrm{tang}}|$$

2.6 $$\mathrm{and}\;a_{\mathrm{lat}}=a-a_{\mathrm{tang}}.$$

where *a* is the fly acceleration, $\hat{v\;}$ is the unit velocity vector and *a*_tang_ is the tangential acceleration, i.e. the component of acceleration parallel to velocity. Lateral acceleration was used as it represents the closest approximation of steering limitation available from the data, and is in keeping with engineering navigational literature [27].

### 2.6. Mapping the outcome of the engagement geometry

We questioned whether the variability in flightpath trajectories could be accounted for by the relative positions of the fly and target at the time of take-off, which we call the geometry of engagement. For this, we ran PN simulations, where the predator always initiated an attack from a ceiling 80 mm above a target that was travelling at 0.79 m s^−1^ from the origin, matching the conditions of real flights. The outcomes (according to metrics detailed in results) of simulated flights were then mapped across the 2D take-off plane over an area encompassing all the recorded take-offs: from 50 mm behind the target to 180 mm ahead and up to 110 mm on either side of it. Simulations were halted if the interceptor came within half a body length (2 mm) of the target. This threshold was arbitrarily chosen but reflects when the target would be within the grasp of the killer fly. Simulations were also halted if the killer fly height dropped 80 mm below the target, reflecting real flies that collided with the floor (electronic supplementary material, movie S1).

Simulation maps were conducted under different kinematic regimes. Three alternative speed profiles were applied to the models, reflecting the mean speed profiles of killer flies taking off from the ceiling, wall, and floor of the arena. Steering limitations (see section above) were also applied to the relevant regime.

### 2.7. Data analysis and statistics

Statistical tests were run on R studio software, v. 1.3.959, and R software, v. 4.0.2 [29]. In this study, mean ± standard error (m ± s.e.) are used as descriptive statistics. Means, medians and standard deviations were calculated using the *pastecs* package. Variance homogeneity was tested using Levene's test, within the *car* package. The significance value (*p*) was 0.05 for all tests, except where *p*-values were adjusted; the circumstances of *p*-value adjustment are specified below.

Correlations between variables were tested using Pearson's *r* tests, implemented in the Hmisc package. Linear models were fitted using the stats package. Comparisons between two datasets with heterogeneous variances were done using a Wilcoxon rank-sum test in the stats package. Multiple groups with heterogeneous variances were compared using a Kruskal–Wallis test, followed by *post hoc* pairwise Wilcoxon comparisons with Hommel's adjusted *p-*values, both in the stats package.

Steering model limitation was validated by a likelihood ratio test run in Matlab R2018b (v. 9.1), using the inbuilt function from the Econometrics toolbox.

## 3. Results

### 3.1. When attacking from the ceiling, killer flies accelerate with similar magnitudes in highly variable trajectories

**Figure 1.**
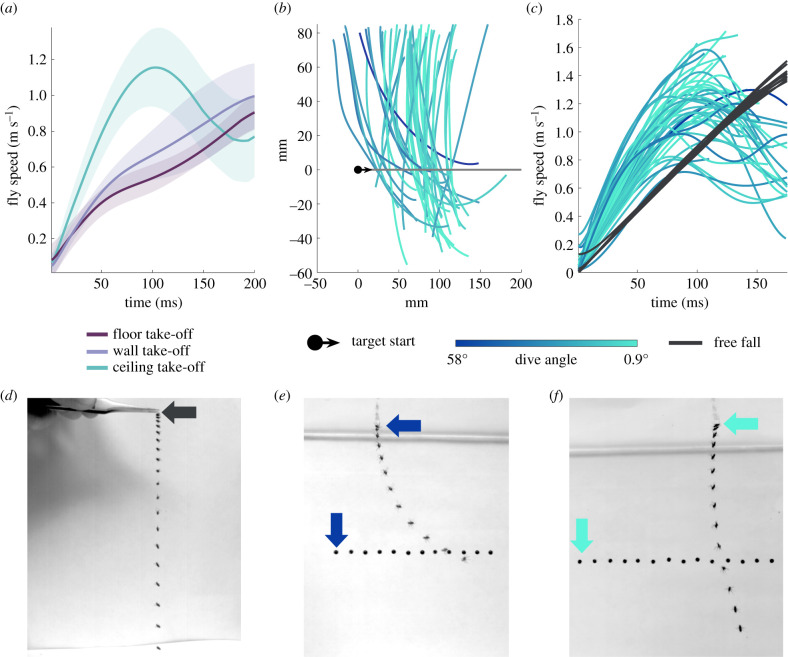
Flies accelerate towards prey when diving downwards, with accelerations independent of dive angle. (*a*) Mean speed of killer flies taking off from the ceiling (*n* = 44, blue), wall (*n* = 12, lilac) or floor (*n* = 9, purple) of the arena. Shaded areas represent ± s.d. (*b*) Side view of the ceiling dives trajectories (blue lines) normalized for target position (black circle) and direction (arrow) at take-off. The intensity of the blue lines has been adjusted to dive angle, with lighter blues indicating more vertical dives, i.e. dives with lower angles. (*c*) Speed of individual killer flies diving towards prey (blue lines, colour-coded for trajectory angle) and anaesthetized killer flies dropped from the same height (*n* = 12, black lines). (*d–f*) Superimposed fly and target positions (shown every 10 ms) during (*d*) a free fall of an anaesthetized fly, (*e*) a dive with a low angle and (*f*) a dive with a high angle. Arrows indicate initial positions in the sequence.

When presented with targets, killer flies (*C. attenuata*) took off by extending the prothoracic legs, initiating wing flapping and detaching the pterothoracic legs from the arena surface, immediately aligning the body towards the target. The location killer flies took off from influenced the kinematics of the attack (figure 1*a*). Killer flies taking off from the ceiling reached significantly higher accelerations (*n* = 44, 17.9 ± 0.703 m s^−2^) than killer flies taking off from the walls (*n* = 12, 10.2 ± 0.332 m s^−2^) or floor of the arena (*n* = 9, 9.84 ± 1.01 m s^−2^, Kruskal–Wallis test, $\chi_{2}^{2}=30.6,$ *p* < 0.001), the latter two not having significantly different accelerations (*p* = 0.920). Interested in how and why such high accelerations are achieved, we analysed in more detail the angles (figure 1*b*) and speeds (figure 1*c*) of attacks initiated from the ceiling, which we termed dives.

When diving, all killer flies tested continuously flapped their wings at an average 303 Hz frequency (±4.11, *n* = 15), for the whole trajectory (electronic supplementary material, figure S3). The mean wing area of killer flies was 2.75 mm^2^ (±0.038, *n* = 20) which, with a maximal stoke amplitude of 145°, means that killer flies' wings operate at an estimated Reynolds number of 276. Despite such a low Reynolds number, the impressive accelerations of diving killer flies led them to reach the height of the target's trajectory in 123 ± 3.96 ms. Although killer flies quickly neared the target's trajectory, most of them (*n* = 41) flew past it and failed to complete the capture (electronic supplementary material, movie S1), meaning the success rate was 7%. We compared the diving flies to anaesthetized flies freely falling (figure 1*d*). The peak accelerations of free falls (*n* = 12, 9.01 ± 0.066 m s^−2^) were significantly lower than the peak accelerations of the dives (Wilcoxon rank-sum test; *W* = 0, *p* < 0.001). The peak accelerations across all dives (min: 11.4 m s^−2^, max: 36.2 m s^−2^) were higher than those from free falls (min: 8.61 m s^−2^, max: 9.32 m s^−2^), suggesting that gravity alone is insufficient to explain the killer fly's accelerations.

Observed dive trajectories were highly variable (figure 1*b*). For example, some paths were profoundly curved, which placed the predator flying almost parallel to the target in the later stages of the attack (figure 1*e*). At the other extreme, during some attacks killer flies flew almost perpendicularly to the target's trajectory (figure 1*f*). We found that the take-off angle in the first 10 ms of the dive could be significantly predicted by the angle of the line-of-sight (LOS), the imaginary line between the fly and its target, at the moment of take-off (*R^2^* = 0.80, *F*_1,42_ = 165, *p* < 0.001). The regression (electronic supplementary material, figure S4) had intercept −17° (s.e. = 3.01, *p* < 0.001) and slope 0.97°/s (s.e. = 0.076, *p* < 0.001). Therefore, although killer flies could manipulate the initial LOS by timing their take-off, they seemed to initiate the attack with a constant 17° lead over their target. The killer fly's dive angle relative to gravity (15.0 ± 1.87°) was not however significantly correlated to the peak acceleration of the dive (Pearson's correlation, *r* = 0.12, *p* = 0.455).

The much higher accelerations recorded in dives from the ceiling compared to other attacks and free falls, together with sustained wingbeat frequencies throughout the dives, indicate that downward attacks were not powered by gravity alone. Killer flies could time their take-off to target position, thereby influencing the angle of their dive. However, dive angle was not correlated with the magnitude of the accelerations, meaning that flies accelerated independently of dive inclination.

### 3.2. Dive kinematics can be replicated using lateral acceleration-limited proportional navigation

**Figure 2.**
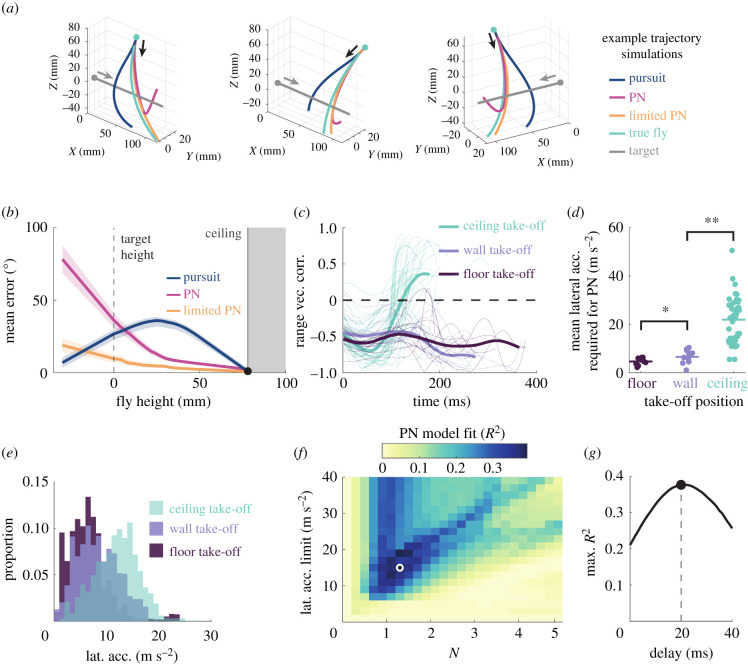
(*a*) Three example simulations of the pure pursuit (dark blue), proportional navigation (pink), and limited proportional navigation (orange) models, compared to the trajectory of real flies (light blue). Arrows indicate direction of movement. (*b*) Angular error of the velocity vector between true flies and each of the model alternatives, plotted against the fly height, 0 being the target height and 80 being the ceiling height. (*c*) Range vector correlation of real killer fly attacks, colour-coded according to take-off positions. Mean lines are given in bold. (*d*) The mean lateral acceleration required to complete a minimal proportional navigation (PN with *N* = 1) towards the target for simulated flights, binned by take-off position. Significance values: *p < 0.05, **p < 0.01. (*e*) Histograms of the lateral acceleration produced by flies starting in different positions. (*f*) The gain fitting map for proportional navigation gain (*N*) and the limit of lateral acceleration, coded by coefficient of determination. White circle denotes peak fit. (*g*) Peak fit of the PN model for time delay.

Differences between attacks from the arena ceiling and attacks from the walls and floor could be due to either a different navigational strategy employed during dives, or to a different interaction of the same navigational strategy with the environment. We implemented models of pure pursuit and PN to compare to the dives performed by real animals (electronic supplementary material, figure S2). Overall, PN was a better predictor of the steering response of real flies, but the rotation axes of PN and pursuit were predominantly aligned. We therefore repeated the analysis on the time points of the data where the two navigational model axes were directionally opposed by over 130°. In this region, PN greatly exceeded the explanatory capacity of pursuit. As found for other attacks, pursuit neither reproduces nor explains the kinematics of killer fly interception, with the simulated fly deviating early from the true flight course and failing to lead the target heading, irrespective of gain tuning (electronic supplementary material, figure S2). This is qualitatively evident from comparing our candidate models (pursuit and PN) to real flightpaths (figure 2*a*), quantified in the angular difference between the models and recorded dives (figure 2*b*).

If killer flies do follow PN even when diving from the ceiling, they should keep a collision course by having negative range vector correlations. We compared the range vector correlation between diving killer flies and flies taking off from the arena wall and floor (figure 2*c*). Although all attacks started with negative range vector correlations, the range vector correlations of attacks from the ceiling turned positive as time progressed, suggesting a failure to maintain an interception course. This failure suggested that the flies either were unable to meet the steering requirements of interception or had lost interest in the target. To quantify the steering effort required, we used lateral acceleration. Using lateral acceleration as a limit of the heading rotation of the fly, we could factor in the effect of the speed at which the fly was travelling (an equivalent lateral acceleration at low speeds will generate greater turning response than at high speeds). We calculated the lateral acceleration required to steer the bare minimum (*N* = 1, below which a collision course is not steered) course towards the target using model steering simulation and the speed of the fly, for flights taking off from the floor, wall and ceiling (figure 2*d*). We found that the required lateral acceleration differed depending on the take-off position (Kruskal–Wallis test, $\chi_{2}^{2}=20.6,$ *p* < 0.001), with higher accelerations in attacks from the ceiling (21.9 ± 1.40 m s^−2^) compared to attacks from the wall (13.5 ± 1.55 m s^−2^, *post hoc* comparisons, *p* = 0.009), whose acceleration was in turn higher than attacks from the floor (9.25 ± 1.00 m s^−2^, *p* = 0.049). We then calculated the lateral acceleration output of the real flies throughout their trajectory and found that the type of surface that flies took off from affected the lateral accelerations they produced (figure 2*e*; Kruskal–Wallis test, $\chi_{2}^{2}=2799,$ *p* < 0.001). In flights from the ceiling, killer flies had higher lateral accelerations (12.1 ± 0.057 m s^−2^) than their wall (8.31 ± 0.074 m s^−2^, *p* < 0.001) and floor counterparts (6.70 ± 0.088 m s^−2^, *p* < 0.001). The mean lateral acceleration requirements of PN with a low *N* = 1 (killer flies previously found to have *N* ≈ 1.5 [7]) are frequently higher than the population of lateral accelerations generated by the fly. This suggests that this may be the limiting factor on the fly.

Parameter estimation (figure 2*f–g*) was applied by sweeping biologically realistic values of lateral acceleration limit (from 0 to 40 m s^−2^), *N* (from 0.1 to 5), and time delay (from 0 to 40 ms). The best fitting value for the lateral acceleration limit (15 m s^−2^) was in keeping with the mean measured fly lateral acceleration of 12 m s^−2^. We secondarily validated the limited model by testing the goodness-of-fit of the pure PN model and the limited PN model, which confirmed that the limited model was still the most probable, despite the additional term (likelihood ratio test, $\chi_{1}^{2}=12.4,$ *p* < 0.001). The angular difference of PN and limited PN from real trajectories (figure 2*a*) shows that the deviation in PN, predominantly occurring in the latter section of the flight, was reduced in the limited PN model. Similarly, simulations comparing limited PN to pure pursuit and PN (figure 2*b*) show that, while pure PN simulations quickly compensate for their overshooting the trajectory, the limited model matches the fly in continuing to overshoot the target downwards.

### 3.3. Killer flies take off in a region that should lead to the shortest trajectory, but are impaired by limited lateral acceleration

**Figure 3.**
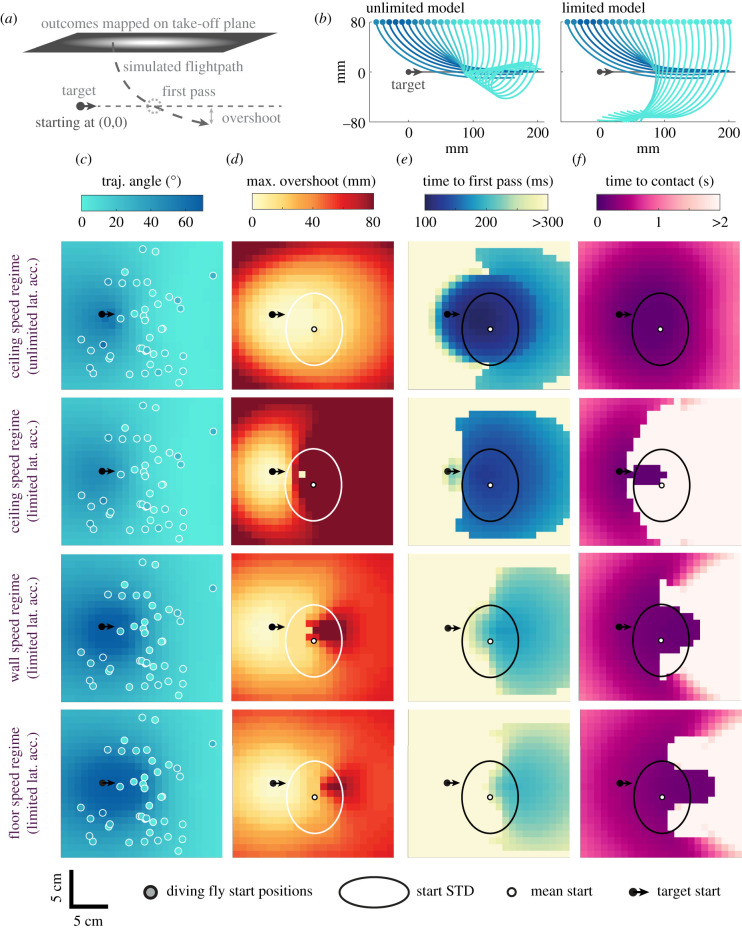
(*a*) An illustration of key terminology for the figure. (*b*) Simulations (blue lines) dropping directly above the target (black dot) and displaced along the target-travel axis are shown for unlimited PN (left) and PN with limited lateral acceleration (right). (*c–f*) Maps of the flight outcomes, by different metrics (columns), of simulations under different lateral acceleration and speed regimes (rows). Coloured dots in (*c*) represent real killer fly take-off positions in ceiling dives (*n* = 44), white dots in (*d–f*) denote mean starting position of true flies, while ellipse denotes ± s.d. of starting positions. Rows represent models with alternative kinematic regimes: unlimited lateral acceleration (top row) and limited lateral acceleration (upper middle row) models with simulated speed equal to the mean speed of killer flies attacking from the arena ceiling, limited lateral acceleration models with the speed of attacks from the wall (lower middle row), limited lateral acceleration models with speed of attacks from the floor (bottom row). Columns display metrics of simulation and real outcomes: (*c*) the resulting dive angle is mapped across the starting plane. Markers give true fly starting positions of diving flies, and measured dive angles. (*d*) The maximum overshot distance of the simulation below the target is mapped across the starting plane. (*e*) The time to first pass is mapped across the starting plane. (*f*) The time taken to contact the target is mapped across the starting plane.

When performing dives, killer flies often missed their targets and overshot (figure 3*a*). The modelled flights with different starting points along the virtual ceiling demonstrate that there is a virtual edge beyond which flies are unable to contact the target on a first pass (figure 3*b*). After this edge, when the lateral acceleration is unlimited, the modelled flies turn a full tight loop and return to a near-collision course with the target. Simulations with limited lateral acceleration also have the same edge, beyond which flies are unable to loop as sharply. In this condition, the flies can only turn onto a collision course with the target once they are far below it.

We took the mean speed profiles of killer flies attacking from the different arena surfaces (ceiling, wall and floor) and tested simulation models starting at the same positions as real killer flies taking off from the ceiling (*n* = 44). We separately tested novel positions, with even spacing across the take-off plane, under the speed regimes of attacks from the ceiling, wall and floor. We found that the trajectory angle (figure 3*c*) was near equally well explained by both the PN models, unlimited (Pearson's correlation, *r* = 0.63, *p* < 0.001) and limited (*r* = 0.64, *p* < 0.001), when given the ceiling-launch speed profile (*R*^2^ = 0.40, *R*^2^ = 0.42, respectively). When given speed profiles of attacks from the walls or floor, the simulated dives were less steep than the real dives, with 93% and 95% of simulations respectively less steep than recorded dives. This demonstrates that speed is a key component of why killer flies' dives are so vertical.

We calculated the model's overshoot up to maximum 80 mm, as in the real arena, for the different conditions (figure 3*d*). Using the unlimited model, minimal overshoot was generally a product of proximity to the target's start, and the flies' starting positions are generally within an area of small overshoot (less than 20 mm). However, when the model has limited lateral acceleration, much of the area ahead of the target generates simulations that overshoot until they hit the floor (80 mm). This area encompasses most of the measured dives. This difference suggests that the modest overshoot of the unlimited model is a product of the model being able to use great lateral acceleration to steer out of the dive, which the limited model and real flies are unable to do. When the models have the kinematic regime as flies taking off from either the walls or floor, the maximum overshoot area is largely absent, bar a much smaller region ahead of the target.

We quantified the point of ‘first pass' by a minimum of distance between target and fly in the first 300 ms of the trajectory (figure 3*e*). We mapped the time to first pass for each modelled starting position and found that the shortest times taken for first pass were close to the flies' mean take-off location. The minimum time to first pass location and time taken varied between models, as minimal times for attacks from the wall and floor were longer and further forward. This is reflected by real killer flies, which first passed the target much sooner when taking off from the ceiling (112 ± 29 ms) than when taking off from the wall (286 ± 61 ms) and floor (311 ± 71 ms). The starting position of real flies was in all cases close to the simulated area of minimal time to first pass.

We also calculated the time to contact in the whole trajectory with an upper limit of 2 s (figure 3*f*). When using the unlimited model, this coincided with the area of minimal time to contact. However, in the limited simulation, a ‘no-hit' region formed in which simulations inevitably overshot targets instead of making contact with them on first attempt. In the simulation with speed regimes of flies taking off from the walls and floors, the ‘no-hit' region shrank, leaving a larger overlap between the areas of minimum time to contact and those of minimum time to first pass.

When attacking from the ceiling, killer flies seemed therefore to take off in the area of minimal time to first pass despite their limitations in lateral accelerations producing a large overshot, which severely affected the time taken to capture the target.

### 3.4. Killer flies maintain wing force magnitude during dives, producing higher aerodynamic power than attacks from the walls or floor of the arena

The navigational model suggests that initiating a dive with some time delay leads to a reduced time to contact, although killer flies prefer to take off in the area of minimum time to first pass. We therefore tested if more vertical trajectories might be energetically more advantageous, requiring less force or power to be completed.

**Figure 4.**
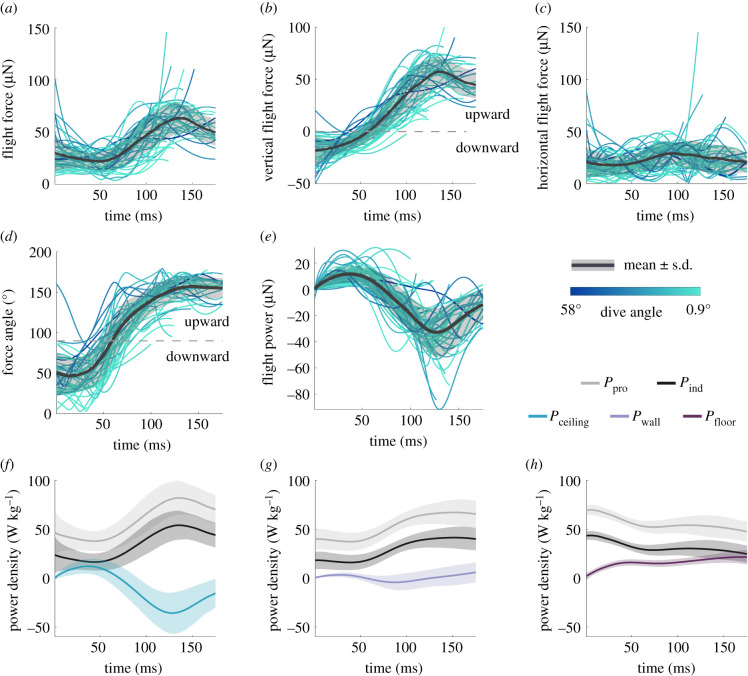
(*a*) Flight force magnitude over time across all dives. (*b*) Vertical component of the flight force. Upward force is shown as positive, downward force as negative. (*c*) Lateral component of the flight force. (*d*) Orientation of the wing force vector over time for all dives. Angles below 90° indicate downward-oriented force vectors, 90° angles indicate horizontal forces, angles above 90° indicate upward-oriented vectors. (*e*) Flight power over time across all dives. (*f*) Flight power density (blue) and wing aerodynamic power densities (grey) of attacks from the ceiling. (*g*) Flight power density (lilac) and wing aerodynamic power densities (grey) of attacks from the wall. (*h*) Flight power density (purple) and wing aerodynamic power density (grey) of attacks from the floor. The aerodynamic power density in *f–h* has been decomposed into profile power (*P*_pro_, grey) and induced power (*P*_ind_, black).

The acceleration of gravity was subtracted from the vertical component of the dive to isolate the acceleration produced by the fly. We multiplied the resulting acceleration by the killer flies' body mass (2.79 ± 0.056 mg, *n* = 45), to calculate the force exerted during the dives. The mean force produced (38.1 ± 1.29 µN) was not significantly correlated with dive angle (Pearson's correlation, *r* = 0.23, *p* = 0.130; figure 4*a*). The force produced initially decreased and subsequently increased during the dives. This further supports the proposition that flies try to maximize force production in the late stages of their attack to match the requirements of PN. This increase came mostly from a change in vertical dive force, which went from downwards, to zero, to upwards (figure 4*b*). By contrast, the lateral flight force was more constant throughout the dive (figure 4*c*), presumably near the fly's maximum capacity.

Wingbeat frequencies were relatively steady throughout the dive (electronic supplementary material, figure S3), suggesting that increases in force production might be achieved in other ways. Mean wingbeat frequencies (303 ± 4.11 Hz, *n* = 15) were also not significantly correlated with dive angle (Pearson's correlation, *r* = −0.1, *p* = 0.731). At the beginning of the dive, killer flies directed their flight force directly downwards, but this vector was subsequently rotated to a horizontal orientation, and upwards in the later stages of the dive (figure 4*d*). The dive angle was significantly correlated to the time at which the force vector change orientation from downwards to upwards (Pearson's correlation, *r* = −0.61, *p* < 0.001), meaning that flies engaged in more vertical dives started directing force upwards later.

We calculated the power produced throughout each dive as the dot product of the flight force and the flight velocity (figure 4*e*). The dive angle did not correlate with either the maximum positive power needed throughout the dive (13.9 ± 1.11 µW, Pearson's correlation, *r* = −0.12, *p* = 0.445), or with the maximum negative power needed to perform the total dive (−36.8 ± 3.05 µW, Pearson's correlation, *r* = −0.04, *p* = 0.801). Similar amounts of flight power were therefore required to perform dives in different directions, suggesting that dives with lower angles are as energetically expensive as dives with higher angles.

Although similar flight force and power are required for dives of different angles, the extreme accelerations produced in more vertical dives could have some advantages, such as the production of higher aerodynamic power. We compared the flight power of attacks starting from the ceiling, walls and floor of arena (figure 4*f–h*). We used total flight power, minus the contribution of gravity, as an indication of the power that could be generated by the wings. We also calculated the profile (*P*_pro_), and induced (*P*_ind_) power density in dives from the ceiling, walls and floor, using the flight force [23]. To compare flight and aerodynamic powers more easily, we recalculated the flight power from our attacks as power density, by dividing the flight power by the flight muscle mass, approximated as 30% (see methods) of the fly's average mass (2.79 mg).

The flight power density reached maximum absolute values of 43.4 ± 3.10 W kg^−1^ when flies took off from the ceiling (*n* = 44), 19.2 ± 2.00 W kg^−1^ when taking off from the walls (*n* = 12), and 28.9 ± 3.95 W kg^−1^ when taking off from the floor (*n* = 9), a difference which was significant (Kruskal–Wallis test, $\chi_{2}^{2}=25.8,$ *p* < 0.001). Moreover, peak power was reached earlier in dives from the ceiling (121 ± 5.62 ms), than in dives from the walls (192 ± 22.9 ms) and floor (184 ± 26.9 ms) of the arena, also a significant difference (Kruskal–Wallis test, $\chi_{2}^{2}=17.4,$ *p* < 0.001). Peak power timing was significantly different between dives starting from the ceiling and attacks from the arena walls and floor, but the latter two did not have a significant difference (*post hoc* comparisons, *p* = 0.002, *p* = 0.005, *p* = 0.804, respectively).

The peak aerodynamic wing power was also higher in dives from the ceiling (152 ± 7.68 W kg^−1^) than in attacks taking off from the walls (120 ± 5.74 W kg^−1^) and floor (115 ± 2.51 W kg^−1^) of the arena. This difference was also significant (Kruskal–Wallis test, $\chi_{2}^{2}=11.1,$ *p* = 0.004, *post hoc* comparisons, *p* = 0.026 and *p* = 0.019, respectively). The peak power of attacks from the arena walls and floor was not however significantly different (*p* = 0.546).

Dives from the ceiling therefore reached higher absolute flight and aerodynamic power than attacks from the walls or floor of the arena. The absolute flight power was also reached sooner in dives from the ceiling.

## 4. Discussion

Our results show that killer flies (*C. attenuata*) respond to targets presented below them by diving down towards them. When taking off from the ceiling of the arena, killer flies reached much higher accelerations than when taking off from the floor or walls, or indeed when in free fall (figure 1). Killer flies beat their wings at similar frequencies independently of dive angle, thereby producing flight forces of similar magnitudes; their speed is therefore determined by both gravity and wing power. During the downward hunts from the ceiling, killer flies averaged an acceleration of 18 m s^−2^, with some individuals reaching accelerations that were larger than 30 m s^−2^. Compared with diving raptors, which achieve accelerations of 6.8 m s^−2^ [5], the killer fly demonstrates itself to be an impressive aerial predator. These accelerations are achieved even though killer flies' wings operate at intermediate Reynolds numbers (approx. 3 × 10^2^). This figure is just above that calculated for fruit flies (*D. virilis*, 200 [30], *D. melanogaster*, 128 [31]) and considerably lower than the dragonfly (700–2400 [32]), highlighting the high viscous forces killer flies experience despite high success rates in the wild [33].

### 4.1. Limited proportional navigation as steering controller

To understand how killer flies control their trajectories during these high acceleration dives, we tested and modelled the control algorithm that could steer killer flies during attacks. We implemented navigational models of PN and pure pursuit. By comparing the axis of the heading rotation for each model to the heading of real flies, we excluded the modified pursuit paradigms with added modulators. Another way of developing interception-type flightpaths is using the principle of deviated pursuit, when the pursuer attempts to steer ahead of the current target position by a set lead. The optimal lead is determined by the velocity of the target relative to the pursuer, and angles other than the optimum will lead to a first pass miss of the target [27]. Deviated pursuit models have been put forward as the underlying algorithm driving the conspecific flights of hoverflies [8], and blowflies (in the elevation plane only) [34], however, in these cases, the pursuer is able to rely on assumed knowledge of target size and speed, information that is unlikely to be available to a generalist predator which hunts a variety of different targets including conspecifics, like killer flies. A final possible interception model could be the forward models proposed for head stabilization in dragonflies, able to correct not only their attitude adjustments (e.g. body roll), but also the parallax of relative translation [28]. Such findings assume internal models of target speed and suggest that dragonflies may have a preconceived flight-plan. We find it unlikely that killer flies use a flight-plan to guide themselves. Killer flies have been demonstrated to rely on an angular size/speed ratio match to determine likely targets, resulting in them chasing after unsuitable targets that match the desired ratio [18]. This finding suggests that killer flies do not rely on target speed assumptions for predation, information that would be necessary to effectively compute the course to the target, therefore leaving only pursuit and PN as suitable models killer flies might use to control steering. Our findings agree with previously published conclusions [7], that killer flies are unlike flies that only chase conspecifics, and do not use pursuit [11,12], but instead use PN with a gain (N) of approximately 1.3 and time delay of approximately 20 ms. Killer flies share this controller with other aerial predators, such as certain robber flies and raptors [7,35].

Our results suggest that PN, combined with the speed of the target, the high speed of diving flies, and the initial geometry, frequently leads to near vertical attacks. While pure PN generally explains the initial steepness of the dive, it differs substantially as the fly approaches the height of the bead, curving back up after overshooting the target (figure 2). Performing this manoeuvre requires extremely large lateral acceleration. After limiting the lateral acceleration of the simulations, the model also overshoots the target in a similar fashion to that observed in the flies, and we find this lateral acceleration limit to be in the region of 15 m s^−2^, although measured fly lateral accelerations do occasionally reach up to 20 m s^−2^. Future work would benefit from closer examination of the flies' orientations during flights. This might give a clearer picture of steering limitations by comparing potential planes of rotation and the limits of force generation. If a link is found between manoeuvre type and limitation, we might expect killer flies to adjust their flight posture to maximize their turning capabilities, e.g. rolling to increase pitch or yaw components during rotation. Lateral acceleration limits may also vary with flight speed, resulting in a more complex picture, beyond the scope of this work to illustrate.

### 4.2. Take-off timing

In our experiments, a trial was started whenever a killer fly was resting anywhere on a surface of the arena. Once the target was detected, killer flies could affect the distance to target at take-off by choosing the timing of their take-off. We cannot know if the flies ‘wait' for the target to approach a specific region, or if their visual target detection system matches a filter for objects passing through a specific region. Previous work [7] has shown that killer flies predominantly take off while their target is coming toward them (as seen in dragonflies [36]), and in the position that would lead to minimal time to contact. Similarly, we found that killer flies take off within the region with minimal time to first pass. This behaviour puts them in the large overshoot region when travelling at the high dive speed, resulting in extremely long times until final contact (figure 3). Further experiments should test dives towards targets travelling at a range of speeds. Such data would allow us to determine whether killer flies adjust their take-off timing according to the target's speed, and to what extent the target overshoot during dives is a product of the specific bead speed used in our experiments (0.79 m s^−1^). In our experiments, killer flies took off with a constant 17° lead over the initial target position when attacking from the ceiling. Further analysis would also test whether this take-off angle is intrinsic to the killer fly or whether it depends on prey speed.

Killer flies could take off later and avoid the large overshoot region. This would lead to attacks that are energetically as expensive, but with reduced time to contact. Therefore, not accounting for the added acceleration of gravity when attacking from above could be seen as maladaptive. Steep dives may not occur in the wild, or at least not often enough to produce a selective pressure, as killer flies have not been observed standing on the underside of leaves. However, killer flies often take off from a variety of elevations relative to their prey, attacking prey flying above as well as below them. Previous modelling of the killer fly's attack strategy has shown that it is highly successful [7], but the effect of chasing prey flying lower than the killer fly was not investigated.

### 4.3. Potential advantages in early take-off

If it were to occur in the wild, a steep dive may not be a hindrance. Firstly, the high speeds and accelerations reached during a dive produce high aerodynamic power on the wings early in the dive, compared to attacks from the side or below the prey (figure 4). This power can be used to assist prey capture especially when chasing erratic prey. It is worth noting that we use a flight muscle ratio [24] and linear relationships with flight force [23] calculated in other *Schizophora* species to calculate aerodynamic power densities, meaning that the absolute values we present might be inaccurate. A thorough investigation of the wing kinematics in flight might confirm or correct the absolute values given in the present article. However, the pattern of the power profiles is unaffected by these inaccuracies, allowing a comparison of these contributions between attacks from different arena surfaces.

Even if the advantage given by high aerodynamic power was irrelevant, a steep dive provides the possibility of an extremely quick capture (electronic supplementary material, movie S2 and figure S5). In the likely case of a miss, the fast-approaching predator is likely to be detected by the prey as a big looming visual stimulus. Looming stimuli cause collision avoidance manoeuvres, which briefly reduce the horizontal velocity of the turning prey as the flight inertia is used to generate angular acceleration [37,38]. The prey therefore trades speed for increased path tortuosity. Killer flies seem to have adapted to catch such evasive prey; their low PN constant has been suggested as a way to optimize erratic target interception [7,35]. By accelerating towards their prey and creating a fast looming stimulus, killer flies might turn their target's escape manoeuvres to their own advantage (electronic supplementary material, movie S3 and figure S6), as some aerial [39] and aquatic [40] vertebrate predators do. This is because, after missing the target in their first dive, killer flies can launch a second attack, at a much reduced distance from the target.

This suggests that there might be another, more subtle reason for not taking into account the direction of gravity in downward attacks. Killer flies are unusual insect predators in that they can chase prey from above, while most other aerial insect hunters chase from below [6,9]. The difficulty of hunting prey against the cluttered ground is exacerbated by the coarse resolution of the killer fly eyes [17], which is poor even relative to other predators with compound eyes [9,41–43]. Under such conditions, diving towards prey at high speed could be beneficial, despite the associated drop in manoeuvrability: by reducing the distance to their targets, killer flies also increase the target's angular size on their retina, which would in turn make it less likely to lose track of it. Killer flies may therefore prioritize not losing sight of their prey, at the expense of flight duration. Thus, an apparently energy-inefficient attack strategy may still be adaptive when considering the need to keep visually tracking the prey.

## 5. Conclusion

Overall, our results show that PN is, in principle, an effective guidance law even when hunting from inverted positions, but not taking into account physical constraints in lateral accelerations decreases its effectiveness. Nevertheless, the effects of not accounting for the direction of gravity during downward dives may hypothetically be compensated by some advantages, such as the quick production of high aerodynamic power in the wings, forcing the potential prey to slow down and manoeuvre, and potentially improving visual tracking.

## Data accessibility

The data and analysis scripts used in this manuscript are part of the Dryad repository (https://datadryad.org/stash) and accessible with the following: https://dx.doi.org/10.5061/dryad.k98sf7m61 [44].
